# Comparative in silico analyses between *Lactiplantibacillus plantarum* and *Bifidobacterium longum* concerning probiotic properties, anti-lipidemic, and anti-diabetic in vitro activities

**DOI:** 10.1186/s12866-025-04062-9

**Published:** 2025-06-06

**Authors:** Asmaa Negm El-Dein, Wafa A. Alshehri, Ashjan F. Khalel, Hassan M. Awad

**Affiliations:** 1 Chemistry of Natural and Microbial Products Department, Pharmaceutical and Drug Industries Research Institute, National Research Centre, Dokki, Giza, Egypt; 2 Department of Biological Sciences, College of Science, University of Jeddah, Jeddah, 21493 Saudi Arabia; 3 Biology Department, University Collage of Aldarb, Jazan University, Jazan, Saudi Arabia

**Keywords:** *Lactiplantibacillus plantarum*, *Bifidobacterium longum*, Genotypic identification, Probiotic properties, In vitro anti-lipidemic and anti-diabetic activities

## Abstract

The gut microbiota plays a crucial role in gastrointestinal health, immune function, and overall well-being. Dysbiosis has been linked to various conditions such as colon cancer, atopic diseases, mental disorders, autoimmune disorders, obesity, and diabetes. This in vitro study aims to assess the safety and functional potential of two probiotic strains, *Lactiplantibacillus (L) plantarum* and *Bifidobacterium (B) longum*, focusing on their anti-lipidemic, anti-diabetic, antioxidant, and probiotic properties. The strains were tested for stress tolerance, including acidic, alkaline, osmotic, oxidative, thermal, detergent, bile salt, and pancreatic enzyme conditions. Both strains exhibited strong resilience, often surpassing the control strain. Their antioxidant activity, measured by radical scavenging ability, was comparable to ascorbic acid, with values of 77% for *L. plantarum* and 92% for *B. longum*. Cholesterol-lowering capacity reached 50% and 49% after 3 days, increasing to 59% and 78% after 7 days, respectively. Hydrophobicity, an indicator of adhesion potential, was approximately 78% for *L. plantarum* and 80% for *B. longum*. Additionally, both strains showed low α-amylase activity (91.65 and 92.33 U/ml), suggesting a potential role in slowing carbohydrate digestion and managing blood glucose levels. Overall, the strains demonstrated favorable safety profiles and promising functional attributes for alleviating hyperlipidemia and diabetes. PCA and heatmap analyses further highlighted *L. plantarum* as the most promising candidate.

**Supplementary Information:**

The online version contains supplementary material available at 10.1186/s12866-025-04062-9.

## Clinical trial

Not applicable.

**Supplementary Information:**

The online version contains supplementary material available at 10.1186/s12866-025-04062-9.

## Introduction

The human gut microbiota is a complex and dynamic ecosystem that plays a critical role in maintaining health. Imbalances in the gut microbiota have been associated with various conditions, such as hypercholesterolemia and diabetes. Probiotic strains like *B. longum* and *L.plantarum* have shown promise in supporting metabolic health and lowering disease risk. Further research and clinical trials are necessary to fully understand and harness their therapeutic potential [1–2].

Gut microbial dysbiosis is closely linked to the development of many diseases, including hypercholesterolemia and diabetes. The use of probiotics, which improve gut microbiota dysbiosis, is highly advocated. However, there is a need for probiotics with stronger anti-diabetic effects [1–3]. Probiotics produce metabolites that promote mucin production and inhibit epithelial cell apoptosis, such as antimicrobial peptides, short-chain fatty acids (SCFAs), and nitric oxide (NO). SCFAs from probiotics maintain the epithelial barrier, promote mucus layer formation, and inhibit the adhesion of pathogenic bacteria [4]. Probiotics enhance the expression of tight junction (TJ) proteins, which play a crucial role in regulating intestinal permeability and maintaining gut barrier integrity. For example, studies have demonstrated that *Lactobacillus rhamnosus* GG can increase the expression of occludin and claudin-1, important tight junction proteins, thereby fortifying the epithelial barrier [5, 6]. Likewise, *Bifidobacterium infants* have been shown to enhance barrier function by boosting ZO-1 expression [7].

In addition to exerting antimicrobial effects directly on microbes, probiotics regulate the inflammatory response through signaling pathways activated in epithelial cells and immune cells either directly or indirectly through metabolite production to restore homeostasis. The human gastrointestinal tract hosts a complex ecosystem consisting of various microorganisms, such as bacteria, fungi, and viruses. The gut microbiota plays a crucial role in nourishing the gut, shaping the immune system, and impacting human health in relation to a range of diseases, including colon cancer, atopic diseases, mental disorders, autoimmune conditions, obesity, and metabolic syndrome. Recent review and experimental studies have emphasized the importance of probiotic-derived metabolites in modulating inflammatory and metabolic pathways for managing diabetes [8–10].

Probiotics, as defined by the World Health Organization, are “live microorganisms that, when consumed in adequate amounts, provide health benefits to the host”. Lactic acid bacteria are extensively researched microorganisms with probiotic properties that have been widely used in various food products. They are known for their ability to survive harsh gastric conditions, adhere to the gut mucosa, and pass through the gastrointestinal tract. Additionally, they exhibit high fermentative activity towards nondigestible food ingredients, which aids in reducing the levels of sugars and other digestible compounds in the lower gastrointestinal tract. Their safety and efficacy have led to their generally regarded as safe (GRAS) status [11]. Two significant diseases affecting quality of life and contributing to rising public health costs are hypercholesterolemia and diabetes. Therefore, it is essential to assess potential probiotics for their effectiveness in addressing these conditions. *B. longum* is a commonly used species in probiotic products known for its probiotic properties and its ability to exhibit anti-lipidemic and anti-diabetic effects in in vitro models. *L. plantarum* is also a versatile species with various probiotic capabilities [1–2].

Probiotics can exert anti-lipidemic effects through multiple mechanisms. They help regulate lipid metabolism by exhibiting various functional properties, such as producing digestive enzymes, enhancing the absorption and utilization of nutrients, lowering cholesterol levels, and exerting anti-inflammatory and immunomodulatory effects. These actions contribute to the prevention and improvement of lipid-related metabolic disorders [12]. Probiotics may also inhibit starch digestion if they lack the ability to produce α-amylase or produce it in only minimal amounts leading to anti-diabetic potential. α-amylase production is strain-specific and not a universal trait of either genus. α-amylase production by *L. plantarum* was reported in various literatures [13–15]. Novel amylase genes were also secreted by certain strains of bifidobacteria [16, 17]. Therefore, probiotic strains with low α-amylase activity are often preferred, particularly for their potential to reduce starch digestion and help manage postprandial blood glucose levels.

*L. plantarum* and *B. longum* are essential members of the stable, non-pathogenic community known as the “healthy” or “normal” gut microbiota. These strains are commonly utilized in the development of functional foods and are frequently present in fermented products like yogurt. When ingested, either through these foods or as free cells, they can provide various health benefits by adhering to the gut lining or transiently interacting with the host’s gastrointestinal tract. Their positive effects are closely tied to regulating host metabolism and have been linked to improvements in several metabolic disorders, including non-alcoholic fatty liver disease, dyslipidemia, insulin resistance, and type 2 diabetes. These effects are achieved through the production and modulation of bioactive compounds such as bacteriocins, conjugated linoleic acid, histamine, hydrogen peroxide, lactate, mannitol, neurotransmitters, and short- or branched-chain fatty acids. By influencing these metabolic pathways, L. plantarum and B. longum play a role in maintaining gut and overall health [3, 18, 19].

Specifically, the novelty of this research lies in the comparative in silico analysis of *L. plantarum* and *B. longum* based on their probiotic potential and health-related properties like anti-lipidemic and anti-diabetic activities, an approach that integrates bioinformatics tools such as heatmap clustering and PCA analysis to predict functional traits based on the experimental data generated in the study. To our knowledge, few studies have systematically compared these two widely used probiotic species using such a comprehensive computational strategy. This approach offers a valuable perspective for targeted probiotic selection based on specific health-related functions. For this instance, this study was designed to compare the probiotic properties, anti-lipidemic, and anti-diabetic activities of *B. longum* and *L.plantarum* using in silico tools.

## Materials and methods

### Sources, isolation and presumptive identification of probiotics

*L. plantarum* was first isolated from Egyptian cholesterol-enriched source (lamb lard) purchased from the market. The sample was collected in sterile plastic containers and stored in a cool box at 4 °C until examination. For bacterial isolation, one gram of the sample was suspended in sterile saline solution (0.85% NaCl), and ten-fold dilutions were prepared [20]. The appropriate dilutions were then inoculated into de Man, Rogosa, and Sharpe medium (MRS) and incubated at 37 °C for 24–48 h in anaerobic jars under anaerobic conditions. A single pure bacterial colony was re-plated and purified on MRS agar plates. The resulting isolate was stored at -80 °C in MRS with 50% (v/v) glycerol as a frozen stock and propagated in MRS broth medium at 37 °C for 24 h before use. LAB were initially identified based on their colony morphology, catalase reaction, Gram staining, and spore formation [21]. The selected colony was confirmed with Gram-positive and catalase-negative reactions, non-spore formation, and non-motility, presumptively identifying it as LAB and selected for further studies. For the catalase reaction, drops of a 3% hydrogen peroxide solution were placed on 24-h-old vegetative cells of each isolate. The presence of bubbles indicated the presence of catalase in the cells (catalase positive) [21]. *B. longum* NRRL B-41,409 was obtained from NRRL, USA.

### Molecular identification of the selected isolate

The genomic DNA (gDNA) of the selected isolate was extracted using a Gene JET Genomic DNA Purification kit, following a modified protocol based on [22]. The DNA concentration was measured using a Nano Drop device, and the gDNA was stored at -20 °C for further analysis. The 16 S rRNA gene was amplified by PCR using specific primers: EuBac-27 F and EuBac1492R. The PCR reaction mixture included PCR Master Mix, primers, and gDNA template. Amplification was performed in a T100 96-well Thermal Cycler with a specific thermal profile involving denaturation, annealing, and extension steps. The quality of the PCR product was assessed by agarose gel electrophoresis with ethidium bromide staining. The PCR products were purified using a Gene JET PCR Purification Kit and sequenced with the EuBac-27 F primer on an ABI 3130 genetic sequence analyzer. Sequencing targeted approximately 1500 bp segments covering the V3 region of the 16 S rRNA gene. The obtained sequences were compared to sequences in DNA databases using BLAST on NCBI (www.ncbi.nlm.nih.gov/blastn). A phylogenetic tree was constructed using MEGA 11.0 software with the neighbor-joining method and bootstrap analysis. The resulting rRNA gene sequences were deposited in the international gene bank.

### Probiotic properties of *L. plantarum* MZ413655 and *B. longum* NRRL B-41,409

#### Stress tolerance

*L. plantarum* and *B. longum* were evaluated for their probiotic characteristics and stress tolerance in the lab. Stress tolerance tests were conducted as per the methodology outlined by [23]. Overnight cultures of the isolates grown in MRS broth at 37 °C were collectedby centrifugation, washed twice with sterile 0.2 M sodium phosphate buffer (pH 7.0), and resuspended in the same buffer to achieve an ODof 1.0 at 600 nm. Various stress conditions were applied, including acidic stress using MRS medium(pH 2.5), glycine-HCl buffer (pH 3.5), alkaline stress using glycine NaOH buffer(pH 9.0), osmotic stress with NaCl, oxidative stress with H_2_O_2_, and heat stress at different temperatures in sodium phosphate buffer. Bacterial cells were exposed to each stress condition for 3 and 6 h at room temperature, followed by sub-culturing in MRS broth and incubation at 37 °C for 24 h. Additionally, detergent stress was tested by inoculating LAB isolates in MRS broth supplemented with various substances such as Tween 80 (0.2%), bile salts (0.5 and 1 g/L), and pancreatic enzymes (1.5 g/L) and incubating at 37 °C for 24 h. Isolates with low bile salt tolerance were cultured in increasing bile salt concentrations until variants capable of growing in 0.1% bile salts for 24 h were obtained. Control experiments involved suspending bacterial cells in sodium phosphate buffer and storing them at 4 °C for an hour, considering them 100% viable to assess viability. All experiments were conducted in duplicate for consistency and accuracy of the results.

#### Cell surface hydrophobicity

The hydrophobicity of *L. plantarum* and *B. longum*, indicating their tendency to adhere to hydrocarbons, was assessed following the method detailed by [24]. In brief, 24 h-old cultures of the strains were collected through centrifugation at 5000 rpm for 10 min at 4 °C, washed twice with 0.1 M sodium phosphate buffer at pH 7.0, and then re-suspended in the same buffer. The cell suspension was standardized to an optical density (OD) of around 1.0 at 600 nm. Subsequently, three milliliters of these bacterial suspensions were mixed with 0.6 milliliters of the non-polar solvent, n-hexadecane (from Merck, Germany), and agitated for 2 min. The mixture was left for an hour at 37 °C to allow separation into layers. The OD at 600 nm of the resulting aqueous phase was recorded. The decrease in absorbance in the aqueous phase was used to calculate the cell surface hydrophobicity (H%) using the formula:

$$\text{H}\%=\:\frac{{OD}_{0}-OD}{{OD}_{0}}\text{*}\:100$$

where OD₀ and OD represent the optical densities before and after extraction with n-hexadecane, respectively. This experiment was duplicated, and the results are presented as mean values with standard deviation.

#### Antioxidant activity of LAB isolates

The DPPH (2,2-diphenyl-1-picrylhydrazyl) scavenging activities of *L. plantarum* and *B. longum* were assessed following the protocol outlined by [25] with slight modifications. The procedure involved mixing equal volumes of ethanolic DPPH solution and bacterial cell suspension, which had been cultured for 24 h and adjusted to a specific optical density (standardized to achieve a final OD600 of 1.0). A control sample was prepared using un-inoculated MRS instead of the bacterial suspension. Ascorbic acid (0.01 g) was used as a positive control. After thorough mixing, the samples were incubated in the dark at 37 °C for one hour. Subsequently, the absorbance of each mixture was measured using spectrophotometry at a wavelength of 517 nm. The scavenging activity was calculated using the formula: #

$$\:\text{\%}\:\text{S}\text{c}\text{a}\text{v}\text{e}\text{n}\text{g}\text{i}\text{n}\text{g}\:\text{a}\text{c}\text{t}\text{i}\text{v}\text{i}\text{t}\text{y}=1-\left(\frac{As-Ab}{Ac}\right)*100$$

Whereas Ab, Ac, and As represent the absorbances of the blank (ethanol and sample), control (DPPH and deionized water), and sample (DPPH and sample) respectively. Following comparative in silico analyses, the most potent phytase-producing isolate will be chosen for molecular identification and subsequent application.

### Safety assessment

#### Blood hemolysis

The safety assessment of *L. plantarum* and *B. longum* focused on their hemolytic activity. To assess hemolysin production, actively growing bacterial cells were cultured on Columbia agar plates enriched with 5% blood from a healthy volunteer. The plates were then incubated aerobically at 37 °C for 24 h. Aerobic conditions were chosen to prevent potential interference with hemolytic activity that could occur under anaerobic conditions. After the incubation period, the plates were examined for signs of hemolysis. Hemolytic activity was categorized based on the appearance of zones around the bacterial colonies. A clear zone of hydrolysis indicated β-hemolysis, while a partial hydrolysis zone with a greenish appearance signified α-hemolysis. The absence of any hemolysis was classified as γ-hemolysis [22]. These observations provided insights into the potential safety profile of the bacterial isolates.

#### Antibiotic susceptibility

The antibiotic susceptibility of *L. plantarum* and *B. longum* was tested against vancomycin (30 µg/disk), ampicillin (10 µg/disk), amoxicillin-clavulanic acid (20/10 µg/disk), penicillin (10 µg/disk), erythromycin (15 µg/disk), azithromycin (15 µg/disk) (Bioanalyse limited, Turkey), and sulphamethoxazole-trimethoprim (1.25/23.75 mg) using the disk diffusion method [26]. The antibiotic discs were placed on MRS medium inoculated with the bacteria and incubated at 37 °C for 24 h. The results were interpreted based on the diameter of the inhibition zone: less than 5 mm indicated resistance(R), 5–15 mm indicated intermediate resistance (IR), and more than 15 mm indicated susceptibility (S) to the antibiotics.

#### Histidine and tyrosine decarboxylase activity (histamine and tyramine formation)

Histidine and tyrosine decarboxylase activity were measured following the method described by [27]. *L. plantarum* and *B. longum* were streaked in duplicate and then incubated for 4 days at 37 °C under anaerobic conditions. Positive histamine and tyramine formation was confirmed by the presence of a purple color around the bacterial colonies.

#### In vitro anti-lipidemic activity as indicated by cholesterol reduction activity (CRA%)

The anti-lipidemic activity was evaluated by measuring the in vitro cholesterol reducing activity (CRA%) of the two strains. Each strain was inoculated into MRS medium supplemented with 1 ml of soluble cholesterol and incubated for 2 and 4 days at 37 °C under anaerobic conditions. The residual cholesterol content in the spent broth was determined using a cholesterol assay kit following the method described by [28]. A control sample of 4 ml of MRS medium with 1 ml of soluble cholesterol was also included. The CRA% was calculated using the formula: CRA % = [(A0– A) / A0] x 100, where A0 is the absorbance of the control at 500 nm and A is the absorbance of the sample at 500 nm.

#### In vitro anti-diabetic activity as indicated by quantitative determination of α‒amylase

The two strains were cultured for 3 days at 37 °C in a modified MRS broth medium containing 2% starch, 2% lactose, and pH 6.2. The amylase activity of each strain was measured using an α-amylase kit (Biodiagnostic Chemical Company, Egypt) following the manufacturer’s instructions. One unit of α‒amylase was equivalent to the amount of enzyme that converts 1mmol of starch to glucose per min at 37ºC and pH 6.2 according to Eq. (1):

$$\:{\upalpha\:}-\text{a}\text{m}\text{y}\text{l}\text{a}\text{s}\text{e}\:\text{a}\text{c}\text{t}\text{i}\text{v}\text{i}\text{t}\text{y}\:\left(\text{U}/\text{m}\text{L}\right)=(\varDelta\:A/min)*765$$

Whereas; ∆A is the change in OD from 1 min to 4 min.

### Statistical analyses

A one-way ANOVA was conducted using Minitab software to determine significant differences between treatments at *P* ≤ 0.05. Principal component analysis (PCA) and a heat map were generated using Origin Lab and GraphPad Prism programs, respectively, to visualize data variation. The experimental results were expressed as mean ± standard deviation (SD).

## Results and discussion

### Molecular identification of the selected LAB strain

#### PCR amplification and primer specificity

Several studies have been conducted to determine the optimal PCR reaction parameters. In the experimental evaluation of PCR amplification performance, the 16 S rRNA gene was amplified using two specific primers: EuBac-27 F and EuBac1492R. The PCR reaction mixture was prepared with PCR Master Mix, primers, and gDNA template, and the reaction was carried out following the conditions outlined in the materials and methods. In this study, the EuBac-27 F and EuBac1492R primer combination was used as a specific primer for *Lactiplantibacillus* sp. The primer was tested through PCR amplification of genomic DNA extracted from the *Lactiplantibacillus* strain. The primers successfully amplified genomic DNA from the isolated *Lactiplantibacillus*, consistent with the findings of [22], which identified these primers as unique to *Lactiplantibacillus* sp. The selected isolate was identified as *L. plantarum* NMP47613ch. The nucleotide sequence has been submitted to the NCBI database (www.ncbi.nlm.nih.gov/blastn) under the accession number MZ413655.

#### Sequencing and phylogenetic analysis

The nucleotide sequence (965 bp) of *L. plantarum* NMP47613Ch was compared to 16 S rRNA gene sequences reported in the GenBank database. The NCBI Blast database (www.ncbi.nlm.nih.gov/BLAST) was utilized to compare the strain *L. plantarum* NMP47613Ch with other *Lactiplantibacillus* strains. *L. plantarum* showed the closest relationship (100% similarity) to *L. plantarum* NMP47613Ch, indicating the highest genetic similarity.

#### Phylogenetic tree construction

**Fig. 1:**
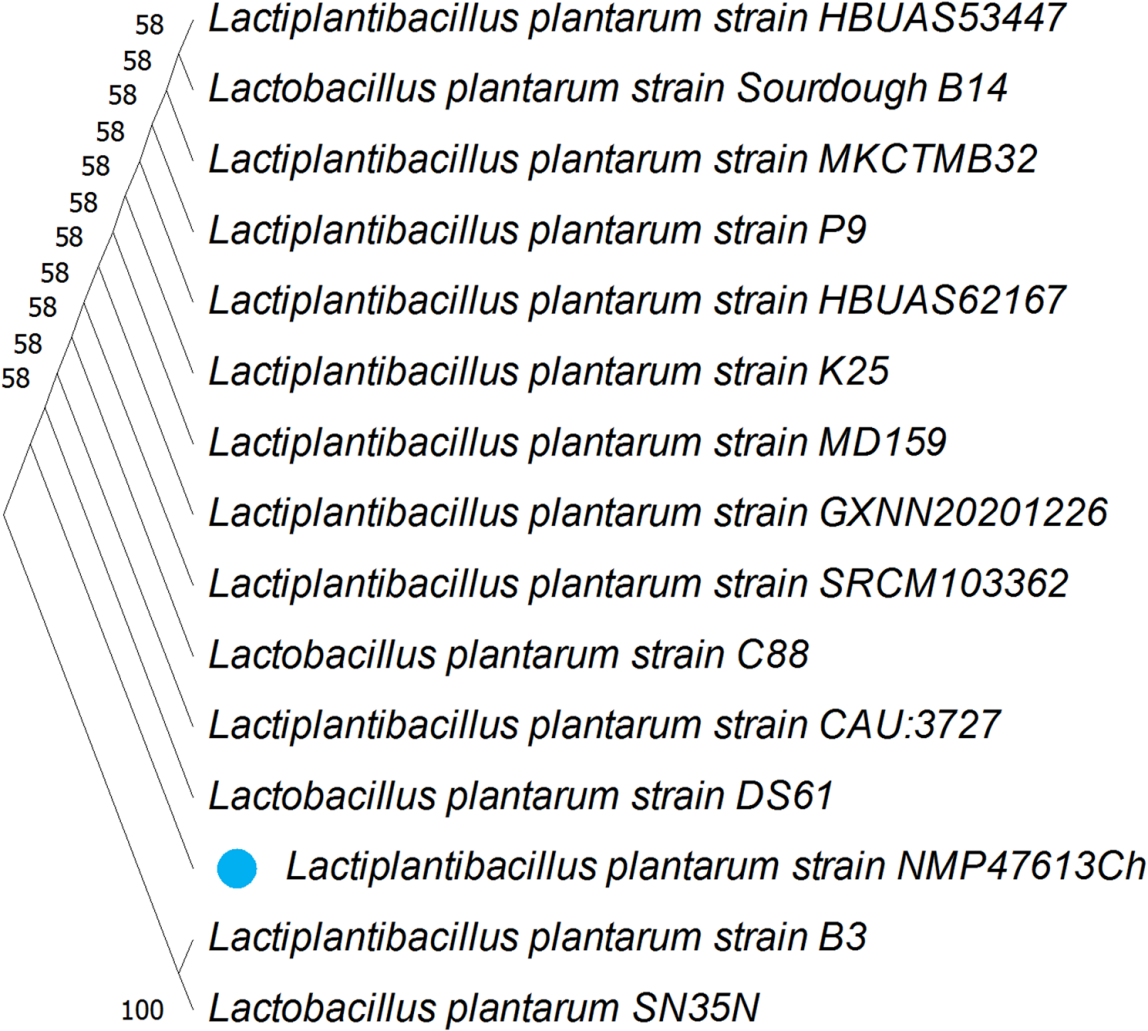
A consensus phylogenetic tree based on 16 S rRNA gene sequences of current recorded *Lactiplantibacillus* strains; beside their corresponding sequences from database. Bootstrap values, more than 50%, of compared algorithms, are indicated at the branch roots

The phylogenetic tree linked here includes all unclassified and classified *Lactiplantibacillus* strains. Distance matrices were utilized to construct the phylogenetic tree through the neighbor-joining method (Fig. 1). The 16 S rRNA sequence of strain NMP47613Ch was found to bear a striking resemblance to *Lactiplantibacillus plantarum*, exhibiting a 100% match.

### Probiotic properties

**Fig. 2:**
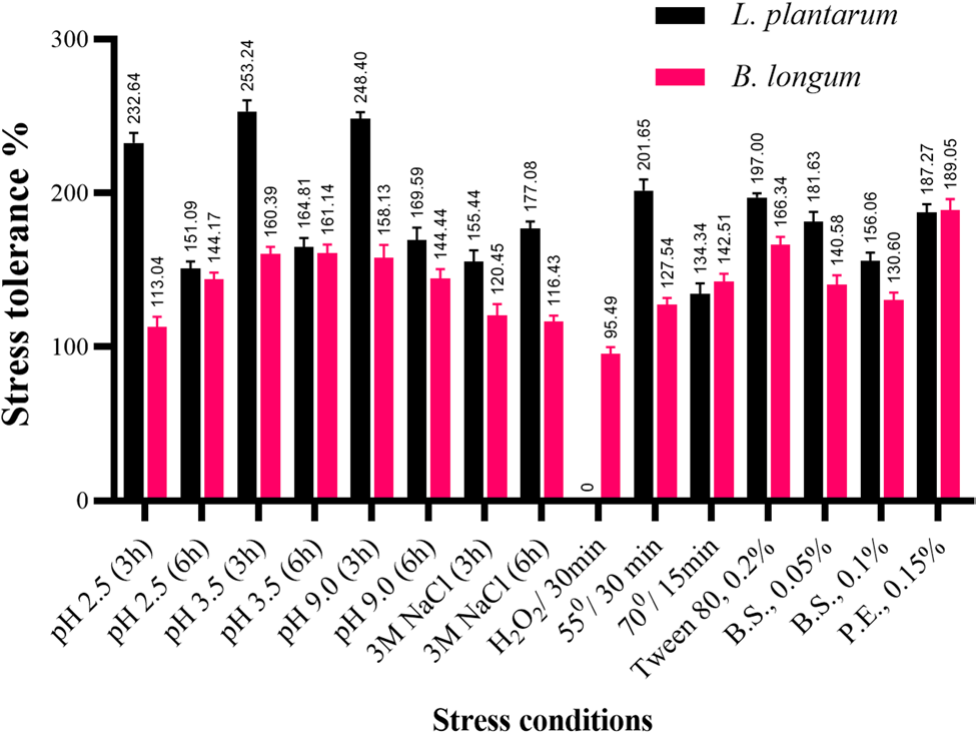
Stress tolerance % of *L. plantarum* and *B. longum* after exposure to different stresses

The acid (acidic and alkaline), bile salts, and pancreatic enzymes tolerance tests were conducted to study the survival rate of the strains under extreme conditions similar to those encountered in the gastrointestinal tract (GIT). Osmotic, oxidative, and temperature stress tests were performed to evaluate the strains’ tolerance to conditions encountered during manufacturing and food processing. After 3 and 6 h of incubation at room temperature (30 ± 2 °C), the stress tolerance percentages (with the control considered as 100%) of the two strains are presented in Fig. 2. The two strains demonstrated potent stress tolerance exceeding the control in most of the conditions tested. *L. plantarum* showed greater tolerance to stress than *B. longum* in most cases, except for oxidative stress where *B. longum* was the only one able to survive (95%). Additionally, *B. longum* (142%) exhibited higher tolerance to exposure to high temperature (70 °C) compared to L. plantarum (134%). Both strains showed similar tolerance to acidic conditions when exposed for a longer period (6 h). A probiotic must withstand exposure to various stressors throughout its lifespan. The stress tolerance of LAB species can vary between strains, making it an essential criterion when selecting strains for probiotics or other industrial uses. Researchers and manufacturers often evaluate these traits to choose specific LAB for different applications [29]. Various species of LAB exhibit bile salt tolerance as they produce bile salt hydrolase, which hydrolyzes bile acids [30]. Previous studies have documented the ability of LAB to withstand these stress conditions and have confirmed an improvement in LAB viability following exposure to stressors [22, 31].

### Antioxidant activity, cell surface hydrophobicity, and cholesterol-reducing ability

**Fig. 3:**
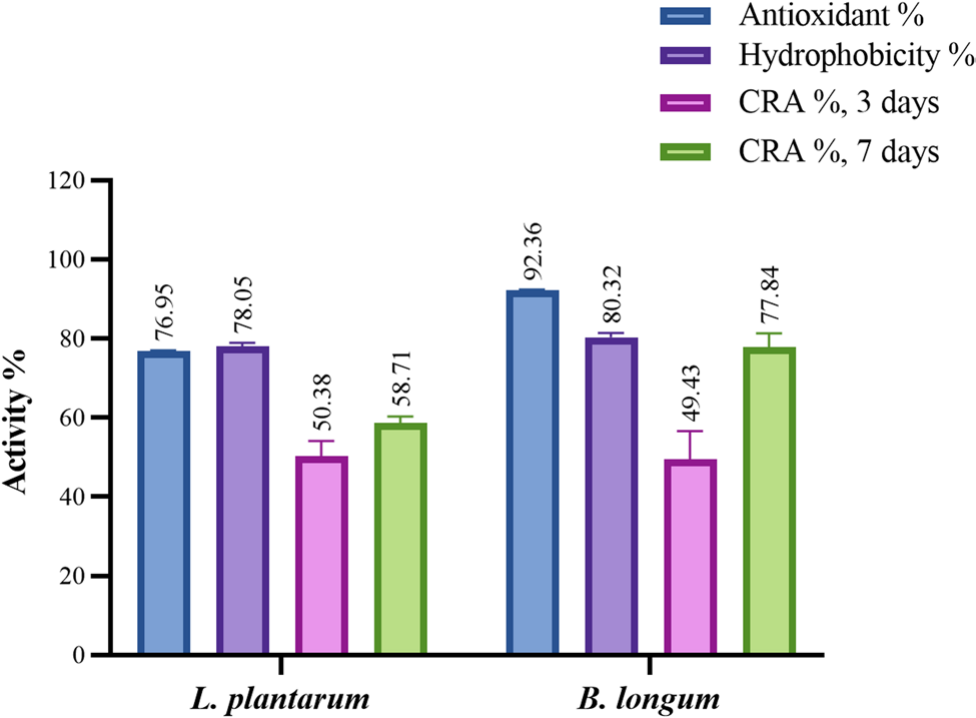
Antioxidant activity, cell surface hydrophobicity, and cholesterol-reducing ability (%)

Compared to the positive control (0.1% ascorbic acid), which demonstrated 100% antioxidant activity, the evaluated CFSs of the two strains exhibited potent antioxidant activity with the superiority of *Bifidobacterium* (77% for *L. plantarum* and 92% for *B. longum*) as illustrated in Fig. 3. LAB exhibit strong antioxidant properties [32]. In Wang’s study, the supernatants from *B. longum* CCFM752, *L. plantarum* CCFM1149, and *L. plantarum* CCFM10 were shown to inhibit ROS production in A7R5 cells, with CCFM10 inhibiting up to 94.6 ± 5.9% of ROS production [33]. Zhao isolated *B. longum* K5 and K10 from infant feces and demonstrated their potent antioxidant activity [34]. Similarly, Son identified *L. plantarum* Ln4 with strong antioxidant activity using a DPPH scavenging assay [35]. Although the exact antioxidant mechanisms of LAB remain unclear, studies have revealed that LAB can produce antioxidant metabolites, scavenge ROS enzymes, enhance host antioxidant enzyme activity, reduce the activity of ROS-related enzymes, and modulate antioxidant signaling pathways in both the host and gut microbiota [36].

Cell surface hydrophobicity (CSH) crucial for microorganisms in determining their ability to attach to or detach from surfaces. In nature, microbes rarely exist as free-floating cells, preferring to form clusters, sometimes called microbial granules [37], which commonly adhere to interfacial surfaces. In environments such as water and soil, microorganisms engage in self-immobilization, with CSH being a crucial factor in this process [38]. The hydrophobic characteristics of microbial surfaces promote adhesion to living and non-living surfaces and facilitate the invasion of host tissues [38–40]. For probiotic strains, CSH serves as a key measure of their ability to colonize the intestines, helping them adhere and persist within the gut. Higher hydrophobicity is linked to better colonization [42], which is further supported by stronger bacterial adherence to hydrocarbons like xylene. The cell surface hydrophobicity (CSH) is based on the hydrophobic components of the outer membrane present in LAB. Hydrophobicity for LAB is crucial in determining cell attachment to epithelial cells and colonization of the strains in the human gastrointestinal tract. The hydrophobicity of the two strains using xylene (a non-polar solvent) was approximately 78% and 80%, respectively (Fig. 3).

Probiotics have been recognized for their cholesterol-lowering effects, largely due to their bile salt hydrolase (BSH) activity [4, 43]. This enzyme enables probiotic LAB to break down or deconjugate bile salts and acids, promoting their excretion or reducing their reabsorption [44], which in turn is associated with a reduction in serum cholesterol levels in the body [45]. In this experiment, the two strains exhibited promising cholesterol-reducing abilities (50% and 49% after 3 days of incubation, and 59% and 78% after 7 days of incubation, respectively), with *B. longum* showing superior results (Fig. 3).

### α-amylase activity

*L. plantarum* exhibited α-amylase activity of about 91.65 ± 1.77 U/ml, while *B. longum* showed approximately 92.33 ± 1.45 U/ml. These levels of activity are considered low, indicating limited anti-diabetic potential as they do not assist in carbohydrate digestion effectively. A promising strategy for managing diabetes mellitus (DM) involves inhibiting the enzymes α-glucosidase and α-amylase, which helps in preventing hyperglycemia. These enzymes are crucial in breaking down complex carbohydrates into simpler sugars in the brush border of the small intestine. By inhibiting them, the absorption of carbohydrates is delayed, leading to reduced postprandial spikes in blood glucose levels. This also helps in controlling blood sugar levels by decreasing insulin secretion after meals [46]. Long-term use of anti-diabetic medications can result in severe side effects, particularly renal impairment. Therefore, altering the gut microbiota to achieve or maintain a healthy balance is recommended for improving overall health. This therapeutic approach has fewer known side effects compared to other available medications. The beneficial live microorganisms introduced into the body for this purpose are commonly referred to as probiotics [47]. Administering probiotics with low α-amylase activity can help modify the gastrointestinal environment and prevent rapid carbohydrate digestion. There is strong evidence suggesting that probiotics can interact with gut-associated lymphoid tissue, inhibit the growth and attachment of harmful pathogens, and influence both mucosal and systemic immunity [48].

### Safety attributes

Safety assessment was performed by measuring hemolytic activity, antibiotic susceptibility, histamine, and tyramine formation. The two strains in this study showed no zone of lysis around the colonies, classified as γ-hemolysis, confirming their safety as non-pathogenic and suitable for probiotic formulations. The strains were susceptible to most tested antibiotics (Table S1 and Table 1), except vancomycin (a common feature of LAB species). They showed intermediate resistance to Amoxicillin-clavulanic acid, and *L. plantarum* also showed intermediate resistance to Azithromycin, which is commonly observed with frequently used antibiotics. In terms of histamine and tyramine production, neither of the strains were capable of producing these compounds, indicating they do not pose a risk of triggering allergic reactions, especially in children.

**Table 1:** Antibiotic susceptibility of the two probiotics

Strain	Vancomycin	Ampicillin	Penicillin	Erythromycin	Amoxicillin-clavulenic	Azithromycin	Sulphamethoxazole/ Trimethoprim
*L. plantarum*	R	S	S	S	IR	IR	S
*B. longum*	R	S	S	S	IR	S	S

R: Resistant (no inhibition zone), IR: Intermediately resistant (inhibition zone 15 mm or less), S: Susceptible (inhibition zone larger than 15 mm)

An important aspect in the evaluation of new probiotics is their safety properties, such as antibiotic resistance. Currently, it is understood that safety properties are highly specific to each strain; therefore, each strain must undergo thorough evaluation [49, 50]. Antibiotic resistance is a significant global health concern that has been rapidly increasing. The main mechanism associated with this issue is the transfer of genes from one microorganism to another. Therefore, probiotics should not carry transferable antibiotic resistance markers [49, 51]. Hemolytic activity is another common virulence factor in pathogenic microorganisms that aids in the acquisition of iron and can lead to anemia in the host [51].

The ability of a probiotic to produce histidine or tyrosine decarboxylase can be problematic, as biogenic amines are linked to negative effects on the neurological, gastrointestinal, and blood pressure systems [52]. The consumption of biogenic amines poses health risks and can have various toxicological consequences. While some LAB species have shown histidine decarboxylase activity [53], the fact that these isolates are unable to produce histamine is advantageous, especially for their use in nutritional products.

#### In silico comparative analyses

From all the above-mentioned in vitro studies, it is difficult to definitively determine which probiotic, *L. plantarum* or *B. longum*, is the most powerful. Therefore, in silico analyses were conducted as they are rapid methods for confirmation and are suitable before proceeding to in vivo evaluations.

**Fig. 4:**
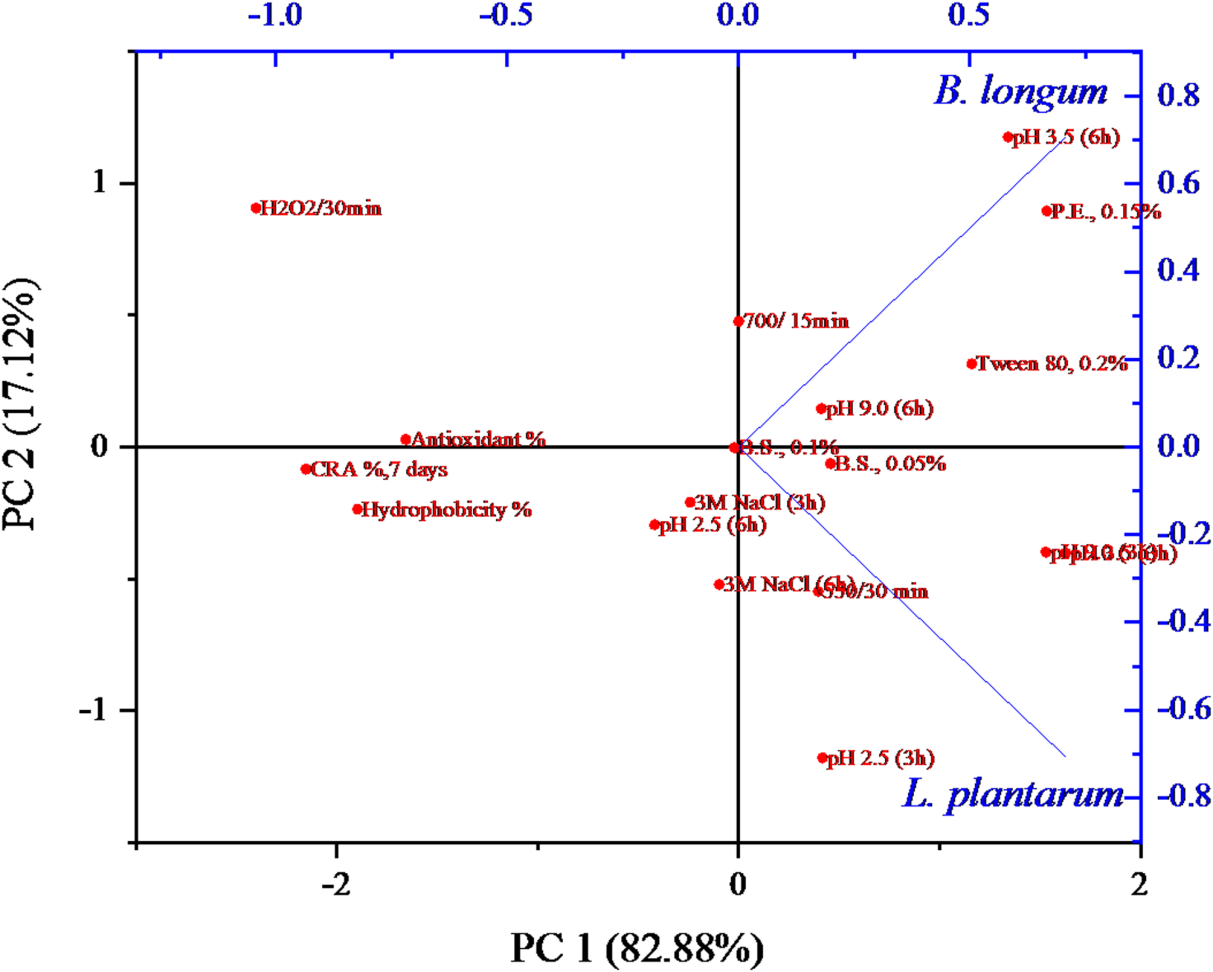
Principal component analysis (PCA) of probiotic properties (acid, alkaline, osmotic, temperature, surfactant, bile, and pancreatic resistances) in addition to antioxidant activity, cell surface hydrophobicity, and CRA (%) of the two probiotics

As depicted in Fig. 4, two principal components (PC1 and PC2) were obtained from the properties, where PC1 and PC2 accounted for 82.88% and 17.12% of the eleven properties (including acid, alkaline, osmotic, oxidative, temperature, surfactant, bile, and pancreatic stresses), in addition to antioxidant activity, hydrophobicity of the cell surface, and cholesterol-reducing ability, respectively. The projections of the two LAB in the PCA plot were differentiated into two quadrants. *B. longum* was positioned in quadrant II, while *L. plantarum* was in quadrant IV. LAB in quadrant IV exhibited a stronger correlation among their properties with respect to PC1 compared to other LAB in quadrant II, which displayed fewer probiotic properties. Therefore, based on the PCA analysis, *L. plantarum* was identified as the more promising probiotic.

**Fig. 5:**
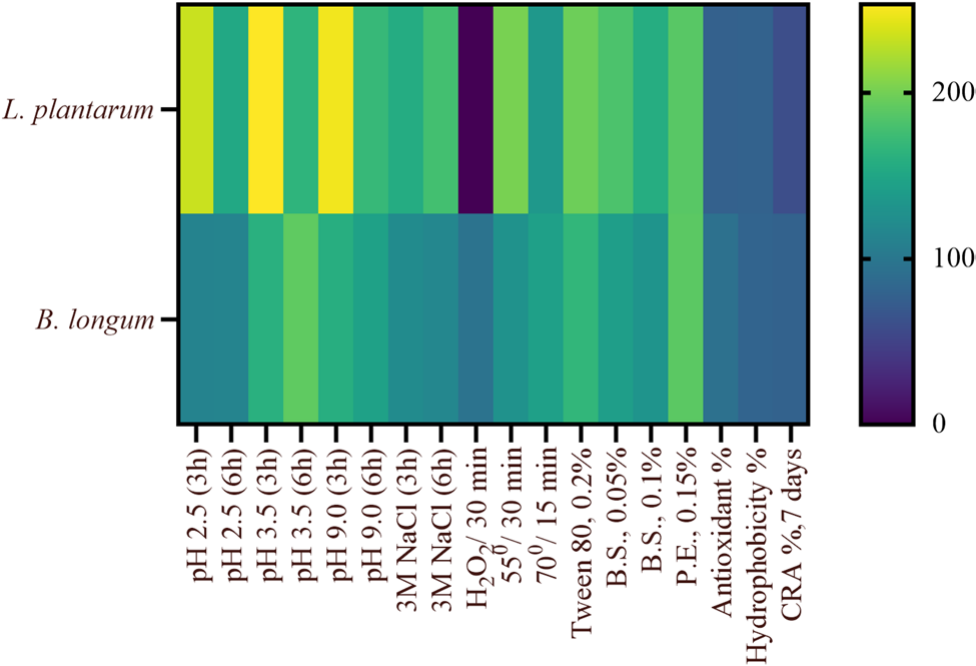
Heat map plot of probiotic properties (acid, alkaline, osmotic, temperature, surfactant, bile, and pancreatic resistances) in addition to antioxidant activity, cell surface hydrophobicity, and CRA (%) of the two probiotics

In contrast to the PCA analysis, the heat map (Fig. 5) visually represents the data variation using different colors. As the percentage value increases, the color or intensity of the color changes (from blue to yellow). In this study, *L. plantarum* demonstrated the most potent probiotic characteristics, as indicated by the intensity of the yellow color.

Considering the overall results of PCA and the heat map, *L. plantarum* was easily identified as the most promising probiotic due to its placement in quadrant IV in PCA and the predominantly yellow color and intensity in the heat map.

Large datasets are becoming increasingly common, but they can be challenging to interpret. Principal Component Analysis (PCA) is a method designed to simplify these datasets, enhance their interpretability, and minimize information loss. It achieves this by systematically capturing more variance in the data through the creation of new, uncorrelated variables. The definition of these new variables is determined by the dataset itself, rather than being created by the analyst from scratch [54]. A heatmap is a visual representation of data in two dimensions that uses color to depict numerical values. This simplifies data interpretation, providing an intuitive grasp of the overall results instead of focusing on specific numerical details.

In conclusion, it can be inferred that *L. plantarum* is a potent and safe probiotic that shows promise as an anti-lipidemic and anti-diabetic agent. Furthermore, it can be beneficially combined with *B. longum*, which also possesses powerful characteristics.

## Conclusion

Hypercholesterolemia and diabetes are prevalent and challenging diseases that have significant impacts on health. Evaluating potential probiotics targeting these conditions is crucial. *B. longum and L. plantarum* are promising probiotic species with anti-lipidemic and anti-diabetic properties. These probiotics have diverse beneficial traits such as enhancing host metabolism and ameliorating metabolic disorders. The study suggests that L. plantarum strains may be superior probiotics compared to *B. longum*, with low α-amylase activity and high anti-lipidemic and antioxidative properties. The findings provide valuable insights into selecting probiotic strains with specific functional attributes, supporting their potential use as biofunctional ingredients in functional foods or dietary supplements. These strains promise for managing metabolic disorders like hyperlipidemia and type 2 diabetes, indicating potential for future clinical validation and application in personalized nutrition and therapeutic interventions. Further research is needed to evaluate their effectiveness in in vivo models.

### Future recommendation

While the current study establishes the probiotic potential of the isolated bacterial strains through in vitro characterization, future research should focus on evaluating their functional efficacy in real-world applications. The isolates’ functional properties, such as acid and bile tolerance, antimicrobial activity, and adherence ability, support their application as potential probiotic candidates in the development of functional dairy products and dietary supplements. This includes incorporating the isolates into food matrices such as fermented dairy or plant-based products and in pharmaceutical preparations to assess their stability, viability, and sensory impact during processing and storage. Additionally, clinical trials and in vivo studies are recommended to validate their health benefits, safety, and mechanisms of action. Such investigations will pave the way for the development of commercially viable probiotic formulations for use in the food and pharmaceutical industries.

## Electronic supplementary material

Below is the link to the electronic supplementary material.

Supplementary Material 1

## Data Availability

Sequence data supporting this study’s findings have been deposited in the NCBI with accession No. MZ413655.
