# Supplementary material for: Comparative in silico analyses between Lactiplantibacillus plantarum and Bifidobacterium longum concerning probiotic properties, anti-lipidemic, and anti-diabetic in vitro activities
Source: BMC Microbiol. 2025 Jun 6;25:356. doi: 10.1186/s12866-025-04062-9 (PMC12143012; doi:10.1186/s12866-025-04062-9)
Supplement: Supplementary file 1 — Supplementary Material 1 [file 12866_2025_4062_MOESM1_ESM.docx]

**Supplementary**

**Table 1.** Breakpoints of sensitivity/resistance (S/R) in the mm inhibitory zone of respective antibiotics based on CLSI, 2018.

| **Sl.No.** | **Antibiotic** | **the inhibitory zone(S/R mm)** |
| --- | --- | --- |
| 1 | Chloramphenicol (C) | (≥18/≤12) |
| 2 | Gentamicin (GEN) | (≥15/≤12) |
| 3 | Clindamycin (CD) | (≥19/≤14) |
| 4 | Ampicillin (AMP) | (≥17/≤14) |
| 5 | Kanamycin (K) | (≥18/≤12) |
| 6 | Tetracycline (TET) | (≥19/≤14) |
| 7 | Vancomycin (V) | (≥17/≤14) |
| 8 | Erythromycin (E) | (≥23/≤13) |
| 9 | Streptomycin (STR) | (≥15/≤12) |
| 10 | Rifampicin (RIF) | (≥20/≤16) |
| 11 | Methicillin (MET) | (≥22/≤4) |
| 12 | Azithromycin (AZM) | (≥13/≤12) |
| 13 | Cefixime (CEF) | (≥21/≤2) |
